# Multiple sulfur isotopes fractionations associated with abiotic sulfur transformations in Yellowstone National Park geothermal springs

**DOI:** 10.1186/1467-4866-15-7

**Published:** 2014-05-28

**Authors:** Alexey Kamyshny, Gregory Druschel, Zahra F Mansaray, James Farquhar

**Affiliations:** 1 Department of Geology and Earth Systems Science Interdisciplinary Center, University of Maryland, College Park, MD 20742, USA; 2 Max-Planck Institute for Marine Microbiology, Department of Biogeochemistry, Celsiusstrasse 1, D-28359 Bremen, Germany; 3 Department of Geological and Environmental Sciences, The Faculty of Natural Sciences, Ben-Gurion University of the Negev, P.O. Box 653, Beer Sheva 84105, Israel; 4 Department of Earth Sciences, Indiana University Purdue University Indianapolis, Indianapolis, IN 46202, USA

**Keywords:** Isotope geochemistry, Sulfur cycle, Sulfide oxidation, Sulfur disproportionation, Multiple sulfur isotopes, Yellowstone national park, Hydrothermal springs

## Abstract

**Background:**

The paper presents a quantification of main (hydrogen sulfide and sulfate), as well as of intermediate sulfur species (zero-valent sulfur (ZVS), thiosulfate, sulfite, thiocyanate) in the Yellowstone National Park (YNP) hydrothermal springs and pools. We combined these measurements with the measurements of quadruple sulfur isotope composition of sulfate, hydrogen sulfide and zero-valent sulfur. The main goal of this research is to understand multiple sulfur isotope fractionation in the system, which is dominated by complex, mostly abiotic, sulfur cycling.

**Results:**

Water samples from six springs and pools in the Yellowstone National Park were characterized by pH, chloride to sulfate ratios, sulfide and intermediate sulfur species concentrations. Concentrations of sulfate in pools indicate either oxidation of sulfide by mixing of deep parent water with shallow oxic water, or surface oxidation of sulfide with atmospheric oxygen. Thiosulfate concentrations are low (<6 μmol L^-1^) in the pools with low pH due to fast disproportionation of thiosulfate. In the pools with higher pH, the concentration of thiosulfate varies, depending on different geochemical pathways of thiosulfate formation. The δ^34^S values of sulfate in four systems were close to those calculated using a mixing line of the model based on dilution and boiling of a deep hot parent water body. In two pools δ^34^S values of sulfate varied significantly from the values calculated from this model. Sulfur isotope fractionation between ZVS and hydrogen sulfide was close to zero at pH < 4. At higher pH zero-valent sulfur is slightly heavier than hydrogen sulfide due to equilibration in the rhombic sulfur–polysulfide – hydrogen sulfide system. Triple sulfur isotope (^32^S, ^33^S, ^34^S) fractionation patterns in waters of hydrothermal pools are more consistent with redox processes involving intermediate sulfur species than with bacterial sulfate reduction. Small but resolved differences in ∆^33^S among species and between pools are observed.

**Conclusions:**

The variation of sulfate isotopic composition, the origin of differences in isotopic composition of sulfide and zero–valent sulfur, as well as differences in ∆^33^S of sulfide and sulfate are likely due to a complex network of abiotic redox reactions, including disproportionation pathways.

## Background

Yellowstone National Park (YNP) contains the highest density of accessible thermal features in the world, with a range of pH and sulfur chemistry conditions uniquely suited to investigating sulfur transformations. YNP is situated above a large magma chamber situated at a depth greater than 8 km and derived from a mantle plume interacting with the North American Plate that extends down 660 km into the mantle transition zone, tilting at a 60 WNW angle [1,2]. The last significant eruption in Yellowstone was 0.6 Ma, with the last known volcanic activity occurring 70,000 years ago; hydrothermal activity is related to the convection of meteoric water driven by heat associated with the cooling magma chamber [3]. The hydrothermal reservoirs in this area are contained in units of alternating ash flow tuff and rhyolitic volcanic deposits, with the largest volumes of water likely contained in the relatively more permeable rhyolitic flows [2-4]. Hydrothermal water discharge in Yellowstone National Park is structurally controlled by fracture intersections associated with both the caldera rim and broader tectonic activity in the area [2,3,5]. Hydrothermal water composition is influenced by a combination of magmatic source fluids and gases, water-rock interactions, and water-vapor phase separation [3,6]. Significant gaseous sulfur is released from magmatic sources in Yellowstone as H_2_S, with relatively little input of SO_2_[7-9]. The remarkable range of chemistries characterizing YNP thermal springs is due to a combination of these processes affecting the deep hydrothermal reservoir and the mixing with relatively oxic shallow meteoric waters.

 Various water types identified at YNP are ultimately produced from a single deep sulfide-rich parent water body. According to Truesdell et al. [10] the chloride and sulfate concentrations and temperature of this water body are 8.74 mmol L^-1^, 115 μmol L^-1^, and 360°C, respectively. Fournier [3] proposed values of 11.3 mmol L^-1^ for Cl^-^ concentration and 335–340°C temperature. In this work we based our quantitative interpretation of the data on the model proposed by Truesdell et al. [10]. This model is based on the observation that composition of most YNP thermal waters can be explained by steam loss during adiabatic cooling of mixtures of a single deep parent water body with shallow, cold waters. Water-rock interactions as well as dilution and boiling alter water composition. Mixing with oxygen-rich subsurface waters leads to oxidation of hydrogen sulfide to sulfuric acid, which was proposed to occur by both abiotic and microbial processes [9,10].

**Figure 1:**
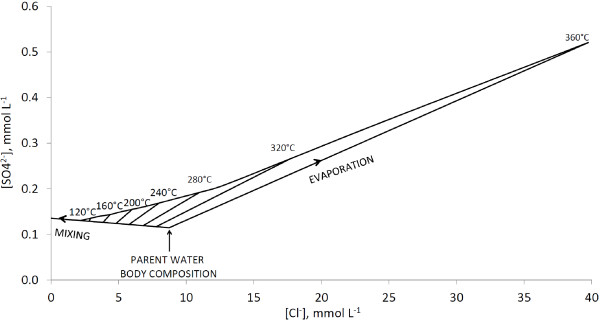
**Sulfate-chloride composition of YNP thermal waters.** Detailed Legend: Sulfate-chloride composition of YNP thermal waters calculated from the model of Truesdell et al. [10]. See explanations in the text.

The process of dilution of the parent water body as well as the process of hydrogen sulfide oxidation can be quantified by chloride to sulfate concentration ratios in the springs and pools [10] (Figure 1). Subsurface waters are diluted with cold, aerated surface water. This process leads to dilution of chloride and a slight increase in sulfate concentrations (up to 115 μmol L^-1^). Further ascent to the surface leads to decompressional boiling in the case that water temperature exceeds 93°C. This temperature corresponds to 2.26 mmol L^-1^ of chloride in the diluted parent water body. Boiling of ascending water with temperatures above 93°C leads to an increase in concentrations of both sulfate and chloride and does not affect chloride to sulfate ratios (Figure 1). Sulfate concentrations, which plot above the array determined by dilution on Figure 1, are due to sulfate produced by surface oxidation of parent water body sulfide.

**Figure 2:**
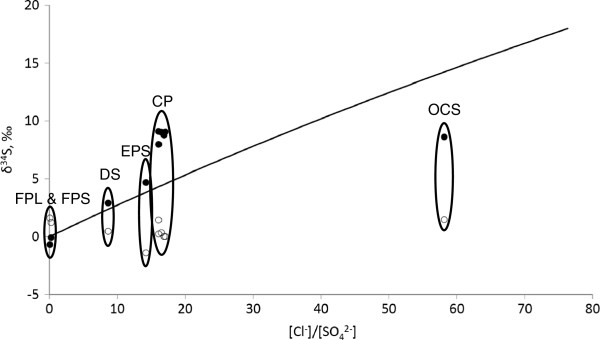
**Sulfur isotope composition in hydrothermal pools as a function of chloride to sulfate ratio.** Detailed legend: Values of δ^34^S for sulfide (open circles) and sulfate (closed circles) in the water column of hydrothermal pools as a function of chloride to sulfate ratio. Solid line depicts δ^34^S of sulfate calculated with the use of the model proposed by Truesdell et al. [10] under assumption that δ^34^S of sulfide is zero.

**Figure 3:**
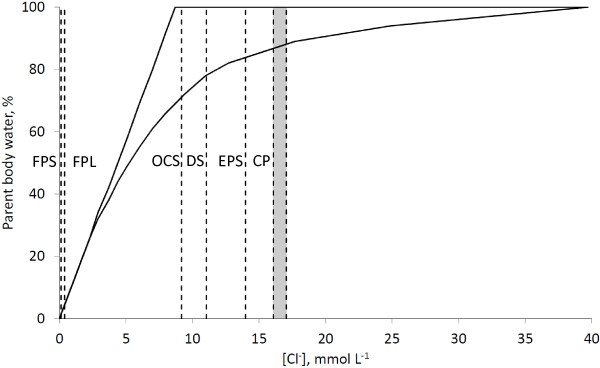
**Scheme of calculation of fraction of parent water body fraction in the thermal waters.** Detailed legend: Scheme of calculation of fraction of parent water body fraction in the thermal waters. Vertical dotted lines depict chloride concentration in the spring waters. Grey area depicts range of concentrations of chloride in the Cinder Pool. Solid lines depict range of parent water body fraction as a function of chloride concentrations calculated from Truesdell et al. [10] model.

We used the Truesdell et al. [10] model to calculate the isotopic composition of sulfate as a function of chloride to sulfate concentrations ratios (Figure 2). In the parent water body sulfate and sulfide are assumed to reach an equilibrium with δ^34^S(SO_4_)-δ^34^S(H_2_S) = 18‰ at 360°C [10]. In shallow water sulfate is produced by non-equilibrium oxidation of sulfide. In this model, both the initial sulfide composition and the fractionation during non-equilibrium oxidation are assumed to equal 0‰. According to this model, spring water with sulfate formed at low temperature has a δ^34^S = 0‰ as all sulfate is assumed to be produced by unidirectional chemical oxidation of hydrogen sulfide with mere zero fractionation. In reality there will be a small fractionation associated with sulfide oxidation as shown at lower temperature by Fry et al. [11]. The model also allows for the fraction of water sourced from the parent water body to be estimated from the chloride concentration of a spring (Figure 3). At chloride concentrations < 2.2 mmol L^-1^, the parent water body is diluted enough that its temperature is below 93°C, and thus dilution is not accompanied by boiling. In this case (FPL and FPS), the exact fraction of parent water body water in spring water can be calculated. If the concentration of chloride is > 2.2 mmol L^-1^, it is a result of dilution and boiling, and only an interval of possible water compositions, rather than exact composition, may be calculated (Figure 3).

In addition to sulfate, some pools, especially those with circumneutral pH, contain high concentrations of thiosulfate [9], the intermediate product of sulfide oxidation by oxygen [12]. Zero-valent sulfur (ZVS) is another common constituent of hydrothermal springs and pools, being more common in acidic sulfate spring ( [9] and references therein). A source of ZVS may be either oxidation of hydrogen sulfide [13,14] or from buried solfataras [15].

Sulfur cycling in geothermal springs is known to be mostly abiotic [9,15] though bacterial sulfate reduction occurs in sediments surrounding these springs. Microbial sulfate reduction rates in sediments of the Cinder Pool have been reported to be ≤2 nmol cm^-3^ day^-1^) [16] and 1 ± 0.27 nmol cm^-3^ day^-1^[17]. Even higher rates of microbial sulfate reduction were reported in the same works for microbial mats in run-off streams. Most of the microorganisms detected in YNP hot springs, including Cinder Pool, rely on hydrogen, rather than on hydrogen sulfide oxidation [18], in agreement with thermodynamic considerations presented in the same publication.

Springs also vary in complexity of sulfur cycling. Some pools discharge in outflow channels, that become oxygenated and cool rapidly, other springs form deep pools, which retain water for long times, resulting in a more complex sulfur cycle. Cinder Pool is the best studied of the pools with a complex sulfur cycle in surface waters [15]. The bottom of the Cinder Pool hosts a lens of molten sulfur. Hydrolysis of molten sulfur generates hydrogen sulfide, thiosulfate and sulfate. The surface of Cinder Pool is partially covered with the hollow “cinders”, which consist of elemental sulfur and pyrite. Oxidation of thiosulfate to tetrathionate with atmospheric oxygen is catalyzed by pyrite [15].

Quantitatively, zero-valent sulfur is one of the most important sulfide oxidation intermediates in the Yellowstone National Park springs and pools. Zero-valent sulfur may be present in natural aquatic systems as four main forms: 1) solid (most probably crystalline, rhombic sulfur), colloidal sulfur [19], sulfur in the form of dissolved polysulfides [19-23], and dissolved (mostly cycloocta-) sulfur [24-26]. Existing analytical techniques for analysis of sulfur speciation in natural aquatic systems [27-29] as well as in understanding thermodynamics of water-sulfur-hydrogen sulfide system [30,31], allows estimation and in some cases, precise quantification of ZVS speciation.

Thiocyanate is another sulfur species of interest in hydrothermal systems. Thiocyanate was detected in geothermal springs [32]. It was also found at the Red Sea Atlantic II brine in concentrations ranging from 23 to 40 μmol L^-1^[33]. Reactions between abiotic hydrogen cyanide and reduced sulfur species were proposed as the source of thiocyanate in the brine. Such reactions are well studied under controlled conditions [29,34-37] as well as in salt marsh sediments [38].

Analysis of quadruple sulfur isotope fractionation is an important novel tool, which was successfully applied to biogeochemical sulfur cycling in modern marine [39] and limnic [40-42] aquatic systems as well as for understanding of sulfur cycling in ancient oceans [43-47]. Small variations in mass-dependent fractionation of multiple sulfur isotopes were proposed to result from complex mass-flow of this element through enzymatic systems [48]. A limited number of studies were performed on multiple sulfur isotope fractionation during sulfate reduction, sulfur disproportionation and sulfide oxidation. Multiple sulfur isotope fractionation during abiotic sulfur cycling in natural aquatic systems was not studied and thus was neglected in previous studies.

In this paper we present a combined study of concentrations of various sulfur species as well as of quadruple sulfur isotope composition of major (sulfate and sulfide) sulfur species and of elemental sulfur in the YNP hydrothermal springs and pools. This study was performed in order to understand multiple sulfur isotope fractionation in the system, which is dominated by a mostly abiotic oxidative part of the sulfur cycle, and to understand how isotopic signals are transformed in sediment. Such data should bear on understanding of sulfur cycling in modern aquatic systems and ancient ocean.

## Results and discussion

Six springs and pools were sampled during a field campaign in May–June, 2010 (Table 1). All sampled pools have temperatures between 72–93°C, but the pH varied from very acidic (1.7 in FPL) to circumneutral (7.4 in OCS) (Table 1). Chloride to sulfate ratio varied from 0.068 in the most acidic pool to 58.2 in the most basic pool. Concentrations of sulfide varied from 16 μmol L^-1^ (EPS) to 121 μmol L^-1^ (DS).

**Table 1:** Temperature, pH and concentrations of sulfur species in YNP springs

**Spring**	**FPL**	**FPS**	**DS**	**EPS**	**OCS**	**CP, 0 m depth**	**CP, 4 m depth**	**CP, 8 m depth**	**CP, 12 m depth**	**CP, 16 m depth**
pH	1.7	3.5	3.4	6.5	7.4	4.7	4.7	4.7	4.7	4.2
T,°C	72	74	76	81	93	75	75	76	77	79
[SO_4_^2-^]	5659 ± 40	520 ± 5	1276 ± 15	984 ± 8	158	557	563	552	564	562
[Cl-]/[SO_4_^2-^]	0.068	0.256	8.65	14.2	58.2	28.9	28.5	29.9	29.9	30.4
[H_2_S]	32.8 ± 0.8	52.3 ± 1.1	121.1 ± 8.3	15.5 ± 1.0	30.7 ± 2.2	28.7 ± 5.4	26.2 ± 0.8	26.1 ± 1.1	22.8 ± 1.0	25.5 ± 0.7
[S^0^]_total_	461 ± 205	91.7 ± 8.6	5.52 ± 3.32	3005 ± 504	0.148 ± 0.063	14.8 ± 2.7	15.1 ± 3.0	13.3 ± 0.5	47.9 ± 49.1	27.2 ± 7.5
[S^0^]_cyanolysis_	1.05 (0.2)	37.5 (40.9)	0.922 (16.7)	26.5 (0.9)	n.a.	3.29 (22.3)	2.95 (19.6)	4.33 (32.5)	4.19 (8.7)	2.78 (10.2)
[S_2_O_3_^2-^]	5.29 ± 0.19	2.94 ± 0.06	3.79 ± 0.36	559 ± 25	4.02 ± 2.34	127.8 ± 4.6	125.6 ± 1.8	125.2 ± 2.5	129.9 ± 3.9	126.1 ± 1.4
[SO_3_^2-^]	0.365 ± 0.090	0.584 ± 0.060	1.47 ± 0.08	2.57 ± 0.70	1.07 ± 0.60	4.49 ± 0.49	4.76 ± 0.07	3.30 ± 1.84	4.49 ± 0.38	4.16 ± 0.39
[SCN^-^]	0.192 ± 0.013	0.164 ± 0.005	0.196 ± 0.013	0.240 ± 0.030	0.791	0.133	0.595	0.138	0.311	0.431

All concentrations are given in μmol L^-1^.

Number in parentheses refer to the percent of total zero-valent sulfur.

### Frying Pan Spring (FPL) and a small spring situated between FPL and the road (FPS)

**Figure 4:**
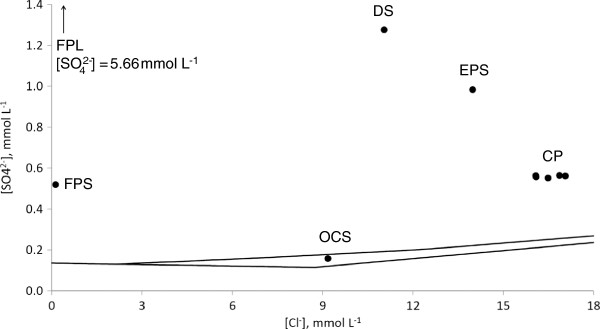
**Chloride and sulfate concentrations in the sampled hydrothermal systems.** Detailed legend: Chloride and sulfate concentrations in the sampled hydrothermal systems plotted on the scheme depicted in Figure 1.

At the springs with the lowest pH values (e.g. 1.7 in FPL, and 3.5 in FPS) concentrations of chloride are low, 0.39 mmol L^-1^ and 0.13 mmol L^-1^ in FPL and FPS, respectively, and concentrations of sulfate are relatively high, 5.66 mmol L^-1^ and 0.52 mmol L^-1^ in FPL and FPS, respectively. Low chloride concentrations indicate a high degree of dilution of the hydrothermal reservoir water body. The parent water body accounts for only 4.4% and 1.5% of water in FPL and FPS, respectively (Figure 3). Low chloride to sulfate molar ratios, in the range of 0.06-0.26, show that the main fraction of sulfate, 98% and 74% in FPL and FPS, respectively, is produced by oxidation of sulfide at shallow depths or at the surface (Table 1, Figure 4). In such a system, dominated by non-equilibrium shallow sulfide to sulfate oxidation in the absence of significant effect of microbial sulfate reduction, isotopic composition of sulfate should be similar to those of sulfide. Indeed, δ^34^S values of sulfide and sulfate in these pools were 1.22-1.59‰ and –0.09--0.69‰, respectively (Table 2). Δ^33^S values of sulfide (-0.017--0.035‰) and sulfate (-0.016–0.025‰), as well as sulfide and sulfate Δ^36^S values, were very similar. These pools were the only ones in which sulfate was more abundant than chloride, although chloride concentrations are relatively high (32.8 μmol L^-1^ and 52.3 μmol L^-1^ in FPL and FPS, respectively). This observation supports model calculations, which show significant sulfide to sulfate oxidation at the shallow depths.

**Table 2:** Isotopic composition of sulfur species

**Spring**	**FPL**	**FPS**	**DS**	**EPS**	**OCS**	**CP, 0 m depth**	**CP, 4 m depth**	**CP, 8 m depth**	**CP, 12 m depth**	**CP, 16 m depth**
δ^34^S, ‰										
Sulfide	1.590	1.215	0.465	-1.404	1.461	0.228	1.428	0.338	0.004	0.005
Sulfur	0.425	0.278	0.374	-0.402	1.069	0.750	1.648	1.338	2.042	1.784
Sulfate	-0.688	-0.092	2.904	4.679	8.604	7.960	9.088	9.018	8.750	9.044
Δ^33^S, ‰										
Sulfide	-0.017	-0.035	-0.030	-0.030	-0.044	-0.064	-0.058	-0.054	-0.041	-0.055
Sulfur	-0.040	-0.038	-0.072	-0.008	-0.063	-0.093	-0.070	-0.058	-0.061	-0.050
Sulfate	-0.025	-0.016	-0.063	-0.031	-0.020	-0.022	-0.026	-0.004	-0.008	-0.053
Δ^36^S, ‰										
Sulfide	0.011	-0.455	0.285	-0.028	0.063	-0.282	0.047	-0.167	-0.103	-0.295
Sulfur	-0.250	-0.294	-0.251	-0.245	0.019	-0.134	-0.059	0.095	-0.199	-0.334
Sulfate	-0.450	-0.200	-0.321	-0.202	0.058	-0.138	-0.046	-0.142	-0.243	0.199

**Figure 5:**
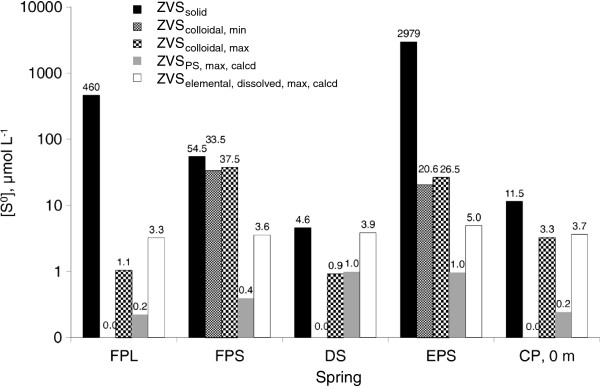
**Zero-valent sulfur speciation in hydrothermal pools.** Detailed legend: Zero-valent sulfur speciation in hydrothermal pools. Pay attention to logarithmic scale of concentration axis.

Analysis of ZVS speciation in the FPL and FPS shows significant speciation differences between these two pools. In the FPL >99% of ZVS is solid dispersed sulfur, and its concentration is 460 ± 205 μmol L^-1^ (Figure 5, note the logarithmic scale). A large standard deviation in the analysis of triplicate samples shows that particles of sulfur in the water column are large enough to add significant variation during the sampling of a 50 ml sample. The colloidal sulfur concentration is ≤1.1 μmol L^-1^. The highest concentration of polysulfidic ZVS calculated to be in equilibrium with sulfide and rhombic sulfur is 0.2 μmol L^-1^. The solubility of rhombic cyclooctasulfur (presented as concentration of atoms of sulfur) in water at the conditions of the FPL pool is 3.3 μmol L^-1^. In the FPS, on the other hand, more than 40% of ZVS is colloidal sulfur, possibly due to the smaller size of the pool, that may lead to smaller retention times for FPS (assuming the fluid inflow/outflow rates between FPL and FPS are not too dissimilar). The total ZVS concentration in FPL was relatively low, 92 ± 9 μmol L^-1^. The maximum polysulfide and dissolved sulfur concentrations in the FPS pool are 0.4 μmol L^-1^ and 3.6 μmol L^-1^, respectively. Comparison of these two springs with similar sulfide concentrations shows that concentrations of ZVS in the pools depend on the surface oxidation processes, rather than on the oxidation of sulfide deeper in the hydrothermal system.

The δ^34^S values of ZVS (0.28–0.43‰) are slightly lower than those of sulfide. This can be interpreted as an absence of equilibration between sulfide and ZVS through polysulfide formation (Eq. 1, [49]) due to the low pH, at which polysulfide anions are not stable with respect to hydrogen sulfide and rhombic S_8_.

The δ^34^S values of ZVS (0.28–0.43‰) are slightly lower than those of sulfide (Table 2). This can be interpreted as an absence of equilibration between sulfide and ZVS through polysulfide formation [49]) due to the low pH, at which polysulfide anions are not stable with respect to hydrogen sulfide and rhombic S_8_ (Eqs. 1, 2 depending on pH).

(1) $$H_{2}S+\left(n-1\right)/8\;S_{8}={{\mathrm{HS}}_{n}}^{-}+H^{+}$$

(2) $${\mathrm{HS}}^{-}+\left(n-1\right)/8\;S_{8}={S_{n}}^{2-}+H^{+}$$

Concentrations of sulfite were 365 ± 90 nmol L^-1^ in FPL and 584 ± 60 nmol L^-1^ in FPS. Concentrations of thiosulfate were 5.29 ± 0.19 μmol L^-1^ in FPL and 2.94 ± 0.06 μmol L^-1^ in FPS. Low concentrations of sulfite and thiosulfate may be explained by fast oxidation of sulfite [50]) and low stability of thiosulfate under low pH conditions [51].

Thiocyanate was detected in both FPL and FPS in sub-micromolar concentrations, 192 ± 13 nmol L^-1^ and 164 ± 5 nmol L^-1^, respectively.

Surface sediment collected near the FPS contained less than 20 μmol kg^-1^ of AVS, 18.5 mmol kg^-1^ of non-ZVS CRS, and 3.15 mol kg^-1^ of ZVS (>10 weight %) (Table 3). Amounts of collected AVS were insufficient for sulfur isotope analysis, and differences in δ^34^S values between ZVS and non-ZVS CRS were less than 1‰ (-0.048‰ and -0.983‰, respectively). Differences between δ^34^S values in the sediment and in the pool water column for both ZVS and non-ZVS CRS were less than 1‰ as well. No significant differences between Δ^33^S and Δ^36^S values of sulfur species in the water column and sediments of the pool were detected. Thus sulfur species in the sediments in the vicinity of the pool are sourced in the pool, and their speciation and isotopic composition are controlled by geochemical processes in the water column of the pool.

**Table 3:** Sulfur speciation and isotopic composition in the surface sediments collected near FPS and FPL

**Spring/species**	**Concentration**	**δ**^**34**^**S, ‰**	**Δ**^**33**^**S, ‰**	**Δ**^**36**^**S, ‰**
		FPS		
[AVS], mmol kg^-1^ (wt% as FeS)	0.019 (<0.01%)	---	---	---
[S-Py], mmol kg^-1^ (wt% as FeS_2_), ‰	18.5 (0.11%)	-0.983 ± 0.025	-0.028 ± 0.007	-0.298 ± 0.140
[S^0^]-tot, mmol kg^-1^ (wt% as S^0^), ‰	3152 (10.1%)	-0.048 ± 0.008	-0.030 ± 0.007	-0.371 ± 0.035
		FPL		
[AVS], mmol kg^-1^ (wt% as FeS), ‰	0.432 (<0.01%)	-1.053 ± 0.021	-0.128 ± 0.038	0.216 ± 0.289
[S-Py], mmol kg^-1^ (wt% as FeS_2_), ‰	227 (1.36%)	0.290 ± 0.016	-0.040 ± 0.001	-0.219 ± 0.140
[S^0^]-tot, mmol kg^-1^ (wt% as S^0^), ‰	6574 (21.1%)	0.701 ± 0.067	-0.043 ± 0.015	-0.139 ± 0.053

All concentrations are given in μmol/L.

The surface sediment collected near the FPL was richer in sulfur species (430 μmol kg^-1^ AVS, 227 mmol kg^-1^ of non-ZVS CRS, and 6.57 mol kg^-1^ of ZVS (>21 weight%) (Table 3). Non-ZVS CRS and ZVS were somewhat heavier than in the FPL, but differences between their δ^34^S values were less than 1‰. AVS in the sediment was lighter than non-ZVS CRS by 1.34‰, and lighter than ZVS by 1.75‰. This difference may result either from AVS oxidation or from bacterial sulfate reduction in the sediment. Microbial sulfate reduction is known to produce isotope fractionations characterized by the difference between δ^34^S values between sulfide and sulfate of –70‰–3‰ [52,53]. Microbial hydrogen sulfide oxidation to sulfate produces fractionation with δ^34^S in the range of –2‰–3‰ [54 and references therein]. Microbial oxidation of sulfide to elemental sulfur produces zero-valent sulfur, which is heavier by 0-3‰ [54 and references therein], although the same or even slightly larger isotope fractionation may be produced by equilibration between sulfur and hydrogen sulfide due to formation of soluble polysulfide species [49].

### Dragon Spring (DS)

DS is another acidic spring with pH of 3.4, but is very different in chemical composition from FPL and FPS (Table 1). The sulfate concentration in this spring is 1276 ± 15 μmol L^-1^, and the chloride to sulfate molar ratio is 8.65. This molar ratio requires that a source of at least 78% of water in the spring is from the parent water body (Figure 3). Shallow sulfide oxidation is, therefore, responsible for production of 85–89% of sulfate in the spring waters (Figure 4). Overall sulfide concentration in the pool is relatively high, 121.1 ± 8.3 μmol L^-1^ (Table 1).

In this spring the sulfate isotope composition is very similar to that calculated using the mixing line based approach of Truesdell et al. [10]: The measured value of δ^34^S is 2.44‰ and the calculated value is 2.38‰ (Figure 2, Table 2). In this pool, the Δ^33^S values of sulfide and sulfate were significantly different (-0.030‰ and–0.063‰, respectively). The origin of this pattern is unclear. While it has been demonstrated that bacterial sulfate reduction can produce variations in Δ^33^S, it also is possible that abiotic disproportionation reactions may produce variations in Δ^33^S.

The total ZVS concentration in the spring is quite low, 5.52 ± 3.32 μmol L^-1^, and only <1 μmol L^-1^ of it is in non-solid, cyanide-reactive form. Up to 4.9 μmol L^-1^ of ZVS may be dissolved in the DS water, 1.0 μmol L^-1^ as polysulfide sulfur and 3.9 μmol L^-1^ as dissolved elemental sulfur. Dissolved and polysulfide zero-valent sulfur pools are apparently not in equilibrium with rhombic sulfur and hydrogen sulfide.

The δ^34^S values of ZVS were close to those of sulfide (0.374‰) (Table 2). This observation confirms an absence of sulfide-polysulfide-sulfur equilibration at low pH values. The Δ^33^S values of ZVS (-0.072‰) were lower than those of sulfide and more similar to Δ^33^S value of sulfate.

The sulfite concentration in the DS water was 1.47 ± 0.08 1.0 μmol L^-1^, and the thiosulfate concentration was 3.79 ± 0.36 μmol L^-1^. Sub-micromolar concentrations of thiocyanate were detected in DS waters as well (196 ± 13 nM).

In the surface sediments collected at the outflow of DS, both acid volatile AVS and non-ZVS CRS (pyrite) contents were too low for isotopic analysis (<400 μmol kg^-1^). Sedimentary ZVS was found to be the main sulfur pool (5.66 mol kg^-1^, 18.1 weight%), and its δ^34^S values were <1‰ lower than the values for ZVS in the water column.

**Figure 6:**
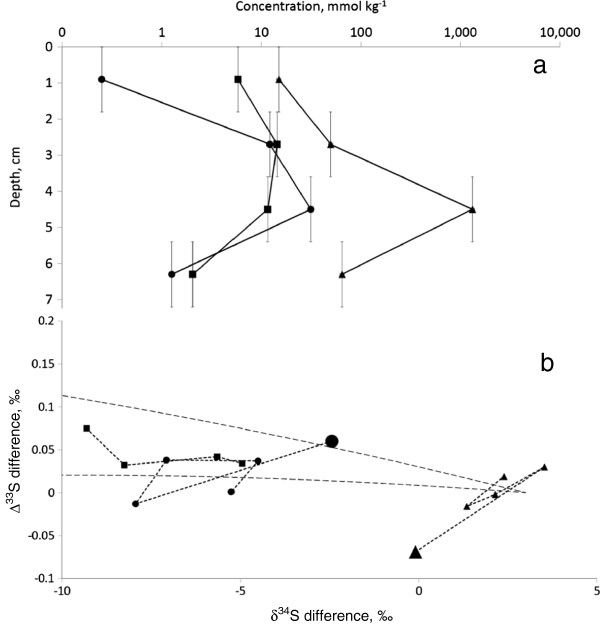
**Concentrations and isotopic compositions of sulfur species in sediment in the vicinity of Dragon Spring.** Detailed legend: Concentrations of AVS (circles), non-ZVS CRS (rectangles), ZVS (triangles) in wet sediment in the vicinity of Dragon Spring **(a)**. Error bars depict width of core slice. Pay attention to logarithmic scale of concentration axis. Difference in sulfur isotope composition between AVS and sulfate (circles), non-ZVS CRS and sulfate (rectangles), ZVS and AVS (triangles) **(b)**. Large dots depict surface sediments. Area confined by dotted lines depicts values for sulphur isotopes fractionation by bacterial sulfate reduction calculated by Farquhar et al. [48] for the [54] model.

In the sediment core taken on the side of the outflow channel 5 m from the spring outflow, the maximum AVS, non-ZVS CRS and ZVS contents were 1.8–5.4 cm below the ground level and were 31.5, 14.4 and 1320 mmol kg^-1^ wet sediment, respectively (Figure 6a). The common trend for all sulfur pools is that δ^34^S values increased with depth and Δ^33^S values were constant (Table 4). In the upper 1.8 cm of the sediment, AVS and ZVS were as much as 1.9–5.5‰ lighter than at the outflow of the spring. The plot of Δ^33^S vs. δ^34^S shows that the direction of sulfur isotope fractionation between reduced sulfur species (AVS and CRS) and sulfate is consistent with the presence of bacterial sulfate reduction, although exact values at some depths differ slightly from those calculated by [48] according to the Brunner and Bernasconi [54] model (Figure 6b). One feasible explanation of the observed trend is a combination of bacterial sulfate reduction with bacterial ZVS disproportionation in the sediment [55].

**Table 4:** Isotopic composition of sulfur species in the sediment core taken in the vicinity of DS

**Depths, cm**	**AVS**			**CRS (non ZVS)**			**ZVS**		
	δ^34^S, ‰	Δ^33^S, ‰	Δ^36^S, ‰	δ^34^S, ‰	Δ^33^S, ‰	Δ^36^S, ‰	δ^34^S, ‰	Δ^33^S, ‰	Δ^36^S, ‰
0.0-1.8	-5.035 ± 0.018	-0.076 ± 0.031	-0.098 ± 0.133	-6.413 ± 0.017	0.012 ± 0.016	-0.461 ± 0.148	-1.509 ± 0.011	-0.046 ± 0.020	-0.383 ± 0.172
1.8-3.6	-4.172 ± 0.015	-0.025 ± 0.019	-0.294 ± 0.271	-5.352 ± 0.004	-0.031 ± 0.013	-0.539 ± 0.153	-2.029 ± 0.006	-0.027 ± 0.015	-0.411 ± 0.144
3.6-5.4	-1.603 ± 0.016	-0.026 ± 0.010	-0.276 ± 0.114	-2.749 ± 0.010	-0.021 ± 0.008	-0.333 ± 0.041	-0.255 ± 0.006	-0.042 ± 0.004	-0.061 ± 0.040
5.4-7.2	-2.356 ± 0.013	-0.062 ± 0.015	-0.283 ± 0.192	-2.051 ± 0.001	-0.029 ± 0.008	-0.224 ± 0.144	0.034 ± 0.006	-0.043 ± 0.014	-0.218 ± 0.069

### Cinder Pool (CP)

**Figure 7:**
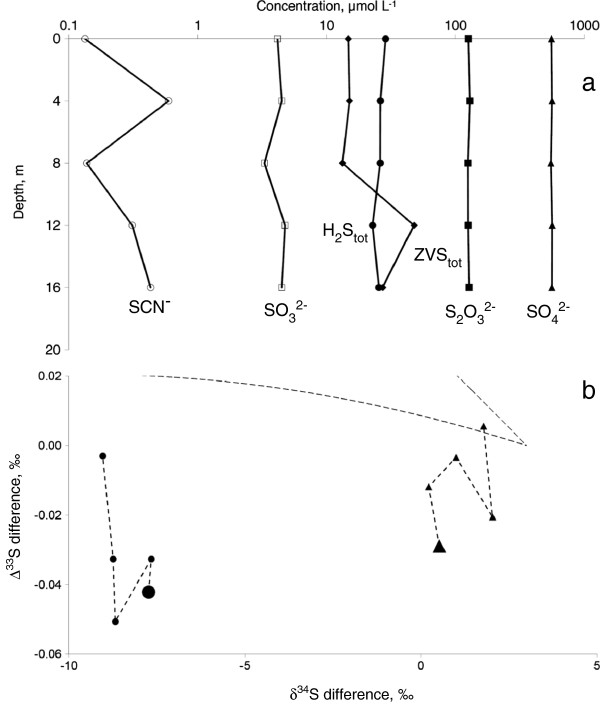
**Depth profiles and isotopic compositions of sulfur species in Cinder Pool.** Detailed legend: Depth profiles of sulfur species in Cinder Pool: **(a)** H_2_S_tot_ stands for total sulfide as detected by the method of [56] and includes protonated and deprotonated free sulfide and acid-reactive metal sulfides. ZVS tot stands for total zero-valent sulfur. See text for details. Pay attention to logarithmic scale of concentration axis. Difference in sulfur isotope composition between sulfide and sulfate (circles), ZVS and sulfide (triangles) **(b)** Large dots depict surface water. Area confined by dotted lines depicts values for sulfur isotopes fractionation by microbial sulfate reduction calculated by [48] for the [54] model.

Cinder Pool is characterized by pH values between 4 and 5 and a much more complex sulfur cycle due to catalytic activity of hollow sulfur-pyrite “cinders”, formed by percolation of gases through a molten sulfur layer on the bottom of the pool. These “cinders” rise to and float on the surface of the pool due to the positive buoyancy of the gas trapped inside the “cinder” [15]. The depth of the pool is c.a. 20 m. We sampled a depth profile, which consisted of 5 points (every 4 m from 0 m to 16 m depth) as well as “cinders” and molten sulfur from the bottom of the pool. The depth profiles suggest that the pool is well mixed and no significant variation in chemical parameters with depth was detected (Figure 7a). The only parameter that varied significantly in triplicate samples was concentration of ZVS.

These findings are in apparent contradiction with results of voltammetric measurements of sulfur species concentrations in CP, which show high variability of sulfide and thiosulfate concentrations with time. Possible explanations of this discrepancy are different spatial and temporal scales of sampling. Tens of milliliters of water were sampled for analysis of concentrations of sulfur species by chromatographic and photometric techniques. Sampling was performed with a peristaltic pump, and we cannot refute the possibility that tubing inlet shifted during the sampling. On the other hand voltammetric measurements were done *in situ*, with the sampling volume surrounding a 100 micron diameter working electrode surface being on the order of hundreds of microliters, and a full scan requiring between 3 and 5 seconds depending on instrument settings. Voltammetry thus records heterogeneity in the water column in ways that sampling for chromatography and spectroscopy did not.

In one of the samples from 12 m depth, the whole cinder was sampled together with the water sample, and the ZVS concentration measured by chloroform extraction was as high as 816 μmol L^-1^ (this sample was omitted from the calculations and graph). In the surface waters of CP, solid sulfur is the main ZVS pool (11.5 μmol L^-1^). Concentration of cyanide-reactive ZVS is only 3.3 μmol L^-1^. As much as 3.5 μmol L^-1^ of ZVS can be solubilized in pool waters as cyclooctasulfur and polysulfides (Figure 5). Thus solid sulfur in the pool is close to equilibrium with dissolved cyclooctasulfur.

Chloride and sulfate concentrations in Cinder Pool have shown that at least 87% of water in the spring is the parent water body (Figure 3) and 54-63% of sulfate is produced by shallow sulfide oxidation. Interestingly, that the δ^34^S values for sulfate (7.96–9.09‰) are 3.5–4.8‰ higher than the values calculated using a mixing line based on the approach of [10]. The reason for this discrepancy may root in an additional fractionation due to the processes involving oxidation of sulfur intermediates (e.g. ZVS, thiosulfate, tetrathionate). Schoen and Rye [57] attributed fractionation between sulfate and sulfide of up to 3.9‰ to abiotic sulfide oxidation to elemental sulfur followed by biological sulfur to sulfate oxidation. Additional support for this explanation may be found in the sulfur isotope fractionation between ZVS and sulfide. In the Cinder Pool water column, δ^34^S values of ZVS are 0.22–2.04‰ higher than δ^34^S values of sulfide, indicating partial equilibration between these two species through polysulfide formation (Table 2). Relatively large differences between sulfide and sulfate Δ^33^S values (up to –0.051‰) (Figure 7b) are likely to be a result of complex sulfur cycling in the pool, involving multiple turnover steps for each sulfur atom [15]. The direction of the Δ^33^S fractionation, opposite to that produced by bacterial sulfate reduction, but consistent with abiotic disproportionation reactions, supports an assumption that it is produced by a complex redox processes network, possibly not enhanced by microbial sulfur reduction in the water column [15].

Concentrations of sulfite in the pool were found to be 3.3–4.8 μmol L^-1^. Concentrations of thiosulfate, 125–130 μmol L^-1^, were higher than in the previously discussed pools, possibly due to a longer water residence time, more complicated sulfur cycle, and less acidic conditions. Concentration of thiocyanate in the pool was as high as 595 nmol L^-1^ (at 4 m depth).

In the “cinders”, the δ^34^S values for non-ZVS CRS (pyrite) and ZVS are 0.597 and 0.879‰, respectively. The values of pyrite and ZVS δ^34^S in the molten sulfur on the bottom of the pool are 0.540‰ and 0.783‰, respectively. These values are quite similar to the δ^34^S values of sulfide and ZVS of dispersed sulfur at the surface of the pool itself (0.228 and 0.750‰, respectively). The Δ^33^S values of non-ZVS CRS and ZVS of these “cinders” and the molten bottom of the pool (-0.036--0.062‰) were slightly higher than the values for sulfide and ZVS at the surface of the pool (-0.064‰ and–0.093‰, respectively).

### Evening Primrose Spring (EPS)

The water column of Evening Primrose Spring was found to be slightly acidic (pH = 6.5). The values of chloride and sulfate concentrations in the EPS show that at least 85% of water in the spring is from the parent water body (Figure 3) and 77-81% of the sulfate is produced by shallow sulfide oxidation. In this spring, the sulfate isotope composition agrees well with a composition calculated from the mixing line reported in [10]: the measured value of δ^34^S is 4.68‰ and the calculated value is 3.91‰. The Δ^33^S values of sulfide and sulfate do not significantly differ. ZVS and thiosulfate were present in this pool in extraordinary high concentrations, 3 mmol L^-1^ and 559 μmol L^-1^, respectively. More than 99% of ZVS is solid sulfur dispersed in the water column of the pool. Concentration of colloidal sulfur in the pool is in the range of 20.6–26.5 μmol L^-1^. Up to 5 μmol L^-1^ and 1 μmol L^-1^ of cyclooctasulfur ZVS and of polysulfide ZVS, respectively, may be present as a soluble form in equilibrium with rhombic elemental sulfur. In contrast to that, the concentration of sulfite was relatively low, only 2.6 μmol L^-1^. Thiocyanate was also detected in this system at 240 nmol L^-1^ concentration.

ZVS δ^34^S values were 1‰ higher than sulfide values, showing that partial equilibration in sulfide–polysulfide–ZVS system was reached. Polysulfides are readily formed at the circumneutral pH and elevated temperatures [31]. On the other hand, only 0.4% of the total ZVS in the EPS passes through a 5 μm pore-size filter. Large sulfur particles have a relatively small surface area, which slows its reaction with sulfide, according to reaction (1). The Δ^33^S value of ZVS was by 0.029‰ lower than the Δ^33^S value of sulfide.

The crust from the side of the pool was found to contain 0.15, 128 and 4134 mmol kg^-1^ of AVS, non-ZVS CRS and ZVS, respectively. AVS δ^34^S values were 2.75‰ lower than the values for sulfide in the pool. The δ^34^S values non-ZVS CRS and ZVS in the crust, equal to–0.242 and–0.244‰, respectively, were similar to the values of ZVS dispersed in the pool water (-0.402‰). The Δ^33^S value of AVS (-0.059‰) was slightly lower than those of non-ZVS CRS and ZVS (-0.024 and–0.026‰, respectively).

### Ojo Caliente Spring (OCS)

In the Ojo Caliente Spring with circumneutral pH (7.4) at least 71% of water in the spring is from the parent water body (Figure 3) and less than 24% of the sulfide is oxidized in the shallow aquifer. In this pool, similar to the Cinder Pool, δ^34^S of sulfate varies from the calculated model value, but it is lighter by 5.6‰ than the calculated values. The Δ^33^S value of sulfate is only slightly (by 0.024‰) higher than that of sulfide. Currently, we have no explanation for the discrepancy between the measured value of sulfate δ^34^S and the one predicted by the model.

ZVS concentrations in the pool (148 nmol L^-1^) were too low to allow speciation or isotope analysis. Concentrations of sulfite and thiosulfate in the OCS were 1.07 μmol L^-1^ and 4.02 μmol L^-1^, respectively. The highest concentration of thiocyanate, 791 nmol L^-1^, was detected in OCS, that is higher compared to ZVS concentrations and only slightly lower than concentrations of sulfite and thiosulfate.

### Synthesis

**Figure 8:**
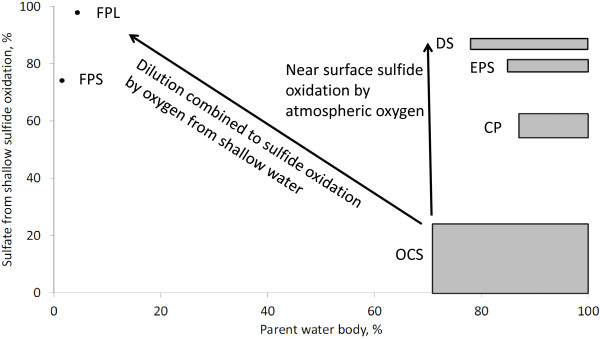
**Plot of sulfate fraction produced by shallow sulfide oxidation *vs*. parent water body fraction.** Detailed legend: Plot of sulfate fraction produced by shallow sulfide oxidation *vs*. parent water body fraction. Increase in sulfate fraction produced by shallow sulfide oxidation accompanied by decrease of parent water fraction points to dilution combined to sulfide oxidation by oxygen from shallow water. Increase in sulfate fraction produced by shallow sulfide oxidation at constant parent water fraction points to near surface sulfide oxidation by atmospheric oxygen.

We propose the following explanation for observed sulfate concentrations in the pools (Figure 8). Low sulfate waters with a high fraction of hydrothermal reservoir water are represented most closely in this study by waters from the OCS. These waters may be diluted by oxic surface waters, a scenario represented by FPL and FPS. Another possibility is that hydrothermal reservoir water reaches the surface with no significant dilution, but has enough time (due to long residence time of water or due to penetration of atmospheric gases into the pool discharge area sediment) to be oxidized by atmospheric oxygen.

**Figure 9:**
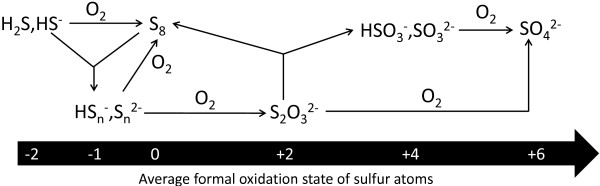
**Scheme of sulfur cycling in the YNP springs and pools.** Detailed legend: Scheme of sulfur cycling in the YNP springs and pools.

The most abundant intermediate sulfur species in all springs, except OCS, was ZVS. Its concentration varied in a wide range from 0.15 μmol L^-1^ in OCS to 3 mmol L^-1^ in EPS. ZVS is possibly produced by the same oxidative pathway as sulfate since concentrations of sulfate and ZVS in springs are positively correlated with an exception of DS (Figures 4 and 5). This may be explained by arguments from [58] and [59] that the first mechanistic step for H_2_S oxidation by O_2_ is formation of elemental sulfur. Elemental sulfur in turn can react via another reduction step to form polysulfide, which in turn oxidizes to intermediates like thiosulfate (stable at intermediate pH) or sulfate (Figure 9). At lower pH elemental sulfur can form by primary oxidation of H_2_S, or disproportionation of polysulfides or thiosulfate.

**Figure 10:**
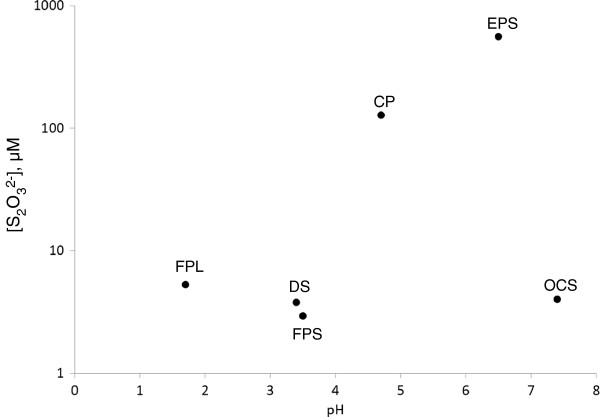
**Concentration of thiosulfate in thermal waters as a function of pH.** Detailed legend: Concentration of thiosulfate in thermal waters as a function of pH. For Cinder Pool data point represents surface waters. Pay attention to logarithmic scale of concentration axis.

The main factors that control concentration of sulfide oxidation intermediates are composition of influx water (especially sulfide concentration), pH, and residence time of water in the spring. Thiosulfate concentrations were in the range of 3–559 μmol L^-1^ (FPS and EPS, respectively). Low thiosulfate concentrations in pools with pH < 3 (FPL, FPS, DS) are the result of fast disproportionation of thiosulfate, which is produced by oxidation of hydrogen sulfide (3) due to reaction (4) (Figure 10).

(3) $$2\;H_{2}S+2\;O_{2}\to S_{2}{O_{3}}^{2-}+2\;H_{2}O+2\;H^{+}$$

(4) $$2\;S_{2}{O_{3}}^{2-}+H^{+}\to {{\mathrm{HSO}}_{3}}^{-}+{{\mathrm{SO}}_{3}}^{2-}+2S$$

The rate of reaction (4) was suggested to be proportional to [H^+^]^1/2^ and [S_2_O_3_^2-^]^3/2^[60] or to [H^+^] and [S_2_O_3_^2-^]^2^[61].

The main fraction of sulfur in all springs was not reactive toward cyanide (>59% in all pools). This means that the most abundant form of zero-valent sulfur in the YNP hydrothermal springs is solid orthorhombic sulfur. We explain it by coagulation and crystallization of colloidal sulfur, which may be produced initially by oxidation of hydrogen sulfide. This explanation is consistent with extremely low fractions of cyanide-reactive sulfur in pools with long water residence times (<1% in FPL and EPS). It is as well consistent with fast (in hours) coagulation and crystallization of colloidal sulfur in the Wadden Sea tidal flat pools that has been recently reported by Kamyshny and Ferdelman [19]. In Cinder Pool, a relatively high fraction of zero-valent sulfur (19 ± 10%) is non-crystalline (e.g. not reactive toward hydrogen cyanide). This may be explained by a) fast turnover of sulfur in the water column of the pool, and b) the fact that one of important zero-valent sulfur reservoir in CP is cinders, which were excluded from sampling for total sulfur concentrations.

The sulfite concentration in all springs was <5 μmol L^-1^ (Table 1). These results agree with data of Zinder and Brock (1977). Sulfite concentrations detected by Zinder and Brock (1977) in CP, EPS, and OCS are 1.9, 9.4 and 0.0 μmol L^-1^, respectively. In this study we detected sulfite in the same systems at 4.5 ± 0.5, 2.6 ± 0.7 and 1.1 ± 0.6 μmol L^-1^, respectively. There are a number of reasons for low sulfite concentration in YNP springs. First, hydrogen sulfide and not sulfur dioxide is a primary volcanic gas in YNP. Second, rates of sulfite oxidation by oxygen are relatively high, especially at low pH [50]. Third, at circumneutral and basic pH, sulfite will react with polysulfides and sulfur to form thiosulfate (reaction opposite to Eq. 4).

The concentration of hydrogen cyanide in the springs was not measured in the spring waters. Possibly it should be very low due to fast conversion of cyanide to thiocyanate by reactions with ZVS species, polythionates and thiosulfate [29,34-37].

Thus thiocyanate may serve as a proxy for hydrogen cyanide concentration in the parent water body. Concentrations of thiocyanate in the pools were in the range of 0.16-0.79 μmol L^-1^ (Table 1). Interestingly, the highest concentration of thiocyanate was measured in the OCS, where it was more than 5 times higher than concentration of ZVS. Reaction between ZVS and hydrogen cyanide may be a contributing factor for low ZVS content in this spring.

Measurements of hydrogen cyanide concentrations in the waters of springs, especially of OCS, are required for understanding of its impact on geochemistry of hydrothermal springs and thiocyanate formation mechanisms.

**Figure 11:**
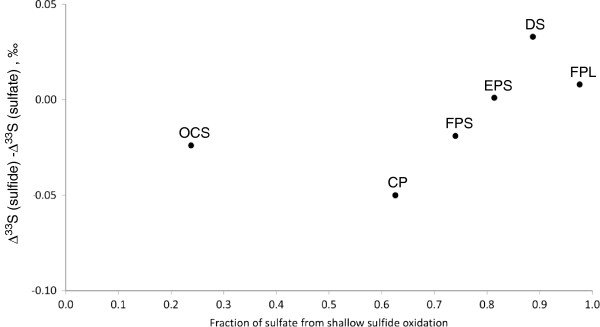
**Δ**^**33**^**S values as a function of fraction of sulfate from shallow sulfide oxidation.** Detailed legend: Difference between ∆^33^S values of sulfide and sulfate as a function of maximum fraction of sulfate from shallow sulfide oxidation (see Figure 4 and related text for explanation).

Sulfur isotope composition of sulfur species is presented in Table 2. The δ^34^S values of sulfide in YNP hydrothermal springs are in the range of 0.0–1.6‰ for all springs. These values are in good agreement with the values for magmatic sulfur [62] and thus confirm magmatic origin of hydrogen sulfide. The ∆^33^S values for sulfide are in the range of –0.041–0.064‰ for CP and –0.003–0.044‰ for all other systems. It is possible that the difference between sulfide and sulfate ∆^33^S values *vs.* the difference between sulfide and sulfate δ^34^S values provides a diagnostic test for biogeochemical pathways of sulfur compounds transformation [39-42,48,63]. Usually multiple sulfur isotope fractionation fingerprints are used to distinguish between bacterial sulfate reduction and its combination with reoxidative sulfur cycling [40,41]. In our case, only in DS sulfate and sulfide did the δ^34^S and Δ^33^S fractionation patterns resemble a bacterial sulfate reduction pattern (Figures 6b and 11). This observation agrees with low bacterial activities in YNP pools, though bacterial sulfate reduction was documented in the water column of Ink Pot Spring (3 nmol SO_4_^2-^ ml^-1^ d^-1^, [64]) and in sediment of CP [16,17]. Relatively large negative fractionation values for Δ^33^S between sulfide and sulfate (-0.05‰) were detected in the Cinder Pool (Figures 7b and 11).

Negative Δ^33^S of sulfide relative to sulfate was found by Johnston et al. [55] in cultures of sulfur disproportionating bacteria and attributed to backflow in the metabolism, which illustrates one case where Δ^33^S is depleted in product sulfide. This case also involved large fractionations, which we do not see here. We propose that complex multi-cycle transformations of reduced sulfur species including disproportionation reactions could be responsible for negative Δ^33^S difference between sulfide and sulfate in these pools and springs. One way to do this would be through disproportionation reactions that yielded fractionated products from an intermediate oxidation state reactant.

**Figure 12:**
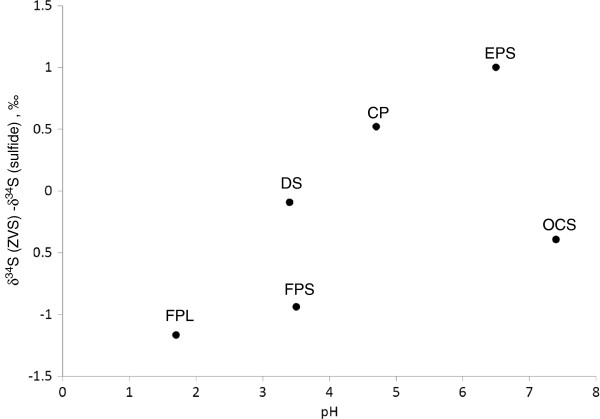
**Difference between δ**^**34**^**S values of ZVS and sulfide as a function of pH.** Detailed legend: Difference between δ^34^S values of ZVS and sulfide as a function of pH for sampled hydrothermal systems. For Cinder pool data point represents surface waters.

In natural aquatic systems at slightly basic pH, the values of zero-valent sulfur are usually isotopically heavier than sulfide due to equilibration through polysulfide formation reactions (Eqs. 1, 2) [49]. In YNP springs zero-valent sulfur was found to be heavier than sulfide in systems with pH of 3.5 and higher, except OC (Figure 12). Equilibrium multiple sulfur isotope effects for abiotic sulfide oxidation were not measured and data from bacterial cultures and lake waters shows that both positive and negative ∆^33^S values can be associated with it [41,42,65]. In the YNP springs, the differences between elemental sulfur and sulfide ∆^33^S values are in the range of –0.042–0.006‰. The origin for this effect is unclear, but it is possibly related to disproportionation reactions involving intermediate sulfur species. Further research is required to clarify this hypothesis.

The Δ^36^S values were suggested to be helpful in interpretation of biogeochemical sulfur cycling [66]. In our work, measurement of Δ^36^S values of sulfur species does not add to understanding of biogeochemical processes due to large standard deviation (±0.2‰). On the other hand, we suggest that performing more precise Δ^36^S measurements in future will possibly enable extracting independent information regarding mechanisms of sulfur cycling in hydrothermal systems.

### Implications for sulfur isotope signatures in modern aquatic systems

Microbial sulfate reduction leads to isotope fractionation with differences in δ^34^S values for sulfide and sulfate ranging between –70‰ and 3‰ [40,52,53]. Absolute values of sulfur isotope fractionation have been shown to decrease with a decrease in sulfate concentration at [SO_4_^2-^] below approximately 2 mmol L^-1^, and values between –30‰ and 3‰ are diagnostic for microbial sulfate reduction at sulfate concentration below 500 μmol L^-1^[67]. In the sampled systems, only in the OCS is [SO_4_^2-^] < 500 μmol L^-1^. Contrary to what is expected in microbially driven process, in this pool we have a relatively high difference, –7.14‰, between sulfide and sulfate. In the pool with highest sulfate concentration (FPL, [SO_4_^2-^] = 5659 μmol L^-1^) fractionation between sulfide and sulfate is represented by δ^34^S value of 2.28‰. Thus, the model of Truesdell et al. [10] provides a sound explanation of the observed δ^34^S values of sulfate and sulfide in waters of hydrothermal springs and pools, and no fractionations that are diagnostic to microbial sulfur transformations are present.

These observations put forward the question regarding possible ways to differentiate between isotope fractionation by chemical processes at high temperatures and microbial processes at low sulfate concentration. We suggest that taking into account fractionation of three sulfur isotopes (^32^S, ^33^S, and ^34^S) of sulfate and sulfide may help in answering this question. A growing body of data is available on fractionation of multiple sulfur isotopes by microbial sulfate reduction, sulfur disproportionation , and sulfide oxidation [48,55,65]. On the other hand, the data on multiple sulfur isotope fractionation by chemical processes involving sulfur species both under controlled conditions and in natural systems is lacking. One of the aims of this work is to shed light on the multiple sulfur isotope fingerprints of complex, mostly abiotic, sulfur cycling.

**Figure 13:**
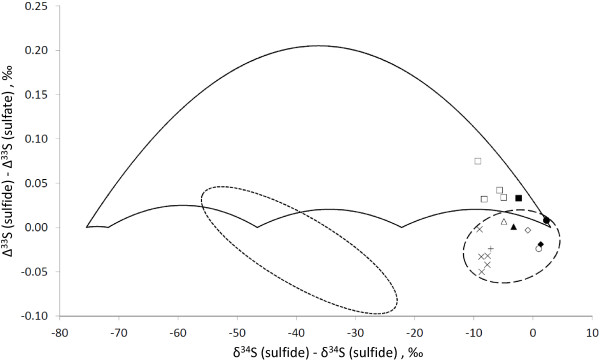
**Sulfide-sulfate Δ**^**33**^**S vs. δ**^**34**^**S values.** Detailed legend: Difference between ∆^33^S values of sulfide and sulfate as a function of difference between δ^34^S values of sulfide and sulfate. Closed circle–FPL water, opened circle–surface sediment near FPL, closed diamond–FPS water, opened diamond–surface sediment near FPS, closed rectangle–DS water, opened rectangles–sediment core taken near DS, Xs–CP water at various depths, opened rectangles–sediment core taken near DS, closed triangle–EPS water, opened triangle–surface sediment near EPS, cross–OC water. Area confined by solid line depicts values for sulfur isotopes fractionation by microbial sulfate reduction calculated by [48] for the [54] model. Area confined by small-dash line depicts values for sulfur isotopes fractionation by microbial sulfur disproportionation [55]. Area confined by large-dash line depicts values for sulfur isotopes fractionation from this work in all springs and pools except DS.

The difference between Δ^33^S values of sulfide and sulfate are positive for microbial sulfate reduction [48,55] and are slightly negative or slightly positive for microbial sulfur disproportionation [55] (Figure 13). In our work isotopic composition of sulfur species of only one system (DS water and sediments) fits the predicted range of values for microbial sulfate reduction. None of the isotopic compositions fit experimental data for microbial sulfur disproportionation. Thus, we suggest that a combination of a) difference in δ^34^S values of sulfide and sulfate that is too low to be interpreted as microbial sulfur disproportionation (≤ 10‰), and b) difference in Δ^33^S values of sulfide and sulfate that is too low to be interpreted as microbial sulfate reduction (≤ 0.00‰), may serve as an indicator for complex abiotic transformations of sulfur species.

Three processes may be responsible for formation of low-Δ^33^S sulfide: 1) deep water body sulfate-sulfide equilibration; 2) reoxidation of sulfide by mixing with oxic shallow waters; 3) geochemical processes during translation of isotopic composition of dissolved sulfide to sedimentary pyrite. As our dataset is quite limited (Figure 13), we can suggest only preliminary conclusions regarding the effects of these processes. In order to explore the first two scenarios, we addressed a connection between a) difference in Δ^33^S values of sulfide and sulfate in the waters of springs and pools; and b) the fraction of sulfate, which is produced by oxidation of sulfide at shallow depths or at the surface (Figure 11). We can see that in waters both unaffected by the surface sulfide oxidation (OCS), and waters affected by a complex surface sulfur cycle (CP) a relatively large negative difference between Δ^33^S values of sulfide and sulfate is detected. Noteworthy in the pools that are the most affected by surface sulfide oxidation (FPL, EPS), a difference between Δ^33^S values of sulfide and sulfate is slightly positive or zero. Thus both equilibration between sulfate and sulfide in the deep water body as well as complex surface sulfur cycling may lead to negative difference between Δ^33^S values of sulfide and sulfate. Unfortunately, our data set is not extensive enough to draw more solid conclusions. Shifts in Δ^33^S values of sulfide and sulfate during signal translation from water column to sediment also does not provide an unequivocal trend. In FPS and EPS sediments, as well as in sediments near the DS (possibly affected by microbial sulfate reduction), we detected a positive shift in the difference between Δ^33^S values of sulfide and sulfate. The opposite trend was observed for the FPL.

Although our work clearly shows that hydrothermal systems affected by shallow sulfide oxidation may produce relatively large negative difference between Δ^33^S values of sulfide and sulfate, our data set is not comprehensive enough to explain mechanisms of formation of this signal.

### Implications for sulfur isotope signatures in geologic record

Multiple sulfur isotope fractionation in Archean ocean was affected not only by biological and abiotic transformations in aquatic systems, but by various arrays of mass-independent isotope fractionations produced by photolysis of atmospheric SO_2_, which originated from volcanic activity [68-71]. A mass-independent isotopic signals were superimposed on mass-dependent isotopic fractionation by microbial sulfate reduction [45,72,73], and possibly by microbial sulfur disproportionation [44]. Other isotopic signals were formed by abiotic and biological transformations involving sulfur sources with mass-dependent sulfur isotope patterns that were not affected by atmospheric chemistry [73]. To make this story even more complicated, the existence of mass-independent fractionation of sulfur isotopes by chemical reactions involving amino-acids and sulfate at high temperatures in the absence of UV radiation was recently discovered [74,75].

Our research provides a framework for evaluation of multiple sulfur isotope signals produced by a complex net of abiotic transformations in a hydrothermal system. We have not observed any mass-independent isotope fractionation between sulfate and sulfide at elevated temperatures during migration of sulfate- and sulfide rich waters to the surface discharge. On the other hand, Δ^33^S values, which were relatively far from zero for mass-dependent fractionation (e.g. -0.128‰ for AVS in the solid samples near FPL, –0.093‰ for zero-valent sulfur in the CP water column, and –0.064‰ for sulfide in the CP water column), were detected. A combination of sulfate-sulfide isotope equilibration at high temperature, phase separation, and oxidative part of the sulfur cycle were found to produce δ^34^S fractionation values between sulfate and sulfide of up to 9‰. This signal may be transformed to even higher fractionation in the solid phase (e.g. in the Evening Primrose Spring difference between δ^34^S values of sulfate and sulfide are 3.3‰ and 4.9‰ in the water column and rocks surrounding the spring, respectively). Thus sulfur isotope fractionation input formed by sulfur transformations in hydrothermal systems may affect interpretation of multiple sulfur isotope fractionation patterns in Archean samples, especially in samples with δ^34^S values close to zero.

Understanding of mass-dependent multiple sulfur isotope fractionation on abiotic sulfur cycling may be especially helpful for the explanation of multiple sulfur isotope fractionation in the sulfide-rich Proterozoic ocean. In the Proterozoic, the concentration of atmospheric oxygen was high enough to prevent formation of mass-independent fractionation of sulfur isotopes, thus allowing detection of small mass dependent multiple sulfur isotope effects produced by microbial and chemical transformations of sulfur species. Existence of an intensive re-oxidative part of the sulfur cycle in the Proterozoic was proposed by results from two sulfur isotopes [76] and multiple sulfur isotope fractionation [43,77,78]. In the works of Johnston and co-authors [43,77,78], it was shown that microbial sulfur reduction in combination with microbial sulfur disproportionation explains the measured triple isotope composition (^32^S, ^33^S, ^34^S) of sulfur species better than microbial sulfate reduction alone. In fact, even the combination of microbial sulfate reduction and microbial sulfur disproportionation failed to explain the composition of some sedimentary sulfide samples with excessively low Δ^33^S values [77,78]. This set of multiple sulfur isotope fractionation data from Yellowstone Natural Park provides observation of fractionation patterns with Δ^33^S_sulfide_ significantly smaller than Δ^33^S_sulfate_ in the natural aquatic system.

## Methods

### Sampling

We sampled six springs with sulfide concentrations >15 μmol L^-1^: I) Frying Pan Spring (FPL; UTM coordinates: 521959 Easting, 4955476 Northing); II) Small spring between Frying Pan Spring and the road (FPS; UTM coordinates: 521959 Easting, 4955476 Northing); III) Dragon Spring (DS; UTM coordinates: 522877 Easting, 4953208 Northing); IV) Evening Primrose Spring (EPS; UTM coordinates: 517696 Easting, 4948724 Northing); V) Ojo Caliente Spring (OCS; UTM coordinates: 512863 Easting, 4934398 Northing); VI) Cinder Pool (CP; UTM coordinates: 523035 Easting, 4953286 Northing). Depth profile containing 5 points as well as isotopic composition of molten sulfur from the bottom of the pool and floating sulfur cinders were sampled at Cinder Pool as well.

All hydrothermal springs and pools water was sampled with a peristaltic environmental pump (Masterflex E/S Variable Speed Water Pump, EnviroTech, CA) and immediately preserved with 1/50 v/v 200 g/L zinc acetate solution for analysis of concentrations of sulfate, sulfide, thiocyanate and ZVS_tot_. Molten sulfur at the bottom of Cinder Pool was as well sampled by a peristaltic environmental pump.

Solid phase was sampled either manually (surface sediments) or by insertion of a tube, constructed by cutting out of the bottom of 60 mL syringe, into the sediment.

### Quantitative analytical methods and calculations

Analyses of total sulfide concentrations (protonated and deprotonated hydrogen sulfide and acid-soluble metal sulfides) were performed by a spectrophotometric technique according to [56], and sulfate concentrations were measured by ion-chromatography. Sulfite and thiosulfate were analyzed by monobromobimane derivatization followed by analysis by HPLC with a fluorescence detector [79]. Derivatization was performed on-site immediately after sampling and samples were immediately frozen and shipped for analyses on dry ice. Speciation of ZVS was measured according to scheme proposed by [38]. Total zero-valent sulfur (ZVS_tot_) was extracted with chloroform and analyzed by HPLC with a UV-visible detector [37]. Sum of all zero-valent sulfur species except solid sulfur (e.g. polysulfidic zero-valent sulfur as well as dissolved and colloidal elemental sulfur, ZVS_cyan_) was analyzed by cyanolysis [29]. Cyanolysis was performed on-site immediately after sampling. Samples were stored at ambient conditions prior to analyses. Individual polysulfide concentrations were not measured as the pH in most pools was too low for application of fast single phase derivatization with methyl trifluoromethanesulfonate [28].ZVS pools presented in Figure 5 were defined and calculated in the following way:

[ZVS_elemental,dissolved,max,calcd_] – concentration of S^0^ in cyclooctasulfur in equilibrium with rhombic sulfur calculated according to thermodynamic constants from [26].

[ZVS_PS,max,calcd_] –concentration of S^0^ in polysulfides in equilibrium with hydrogen sulfide and rhombic sulfur calculated according to thermodynamic constants from [31]. Each polysulfide chain is supposed to have composition S^2-^S^0^_n-1_.

[ZVS_dissolved,total,calcd_] – maximum possible concentration of ZVS in forms of cyclooctasulfur and polysulfides.

$$\begin{array}{ll} \left[{\mathrm{ZVS}}_{\mathrm{dissolved},\mathrm{total},\mathrm{calcd}}\right]= & \left[{\mathrm{ZVS}}_{\mathrm{elemental},\mathrm{dissolved},\max,\mathrm{calcd}}\right]\\ & +\left[{\mathrm{ZVS}}_{\mathrm{PS},\max,\mathrm{calcd}}\right] \end{array}$$

[ZVS_solid_] – concentration of dispersed solid (rhombic) sulfur in the water column.

$$\left[{\mathrm{ZVS}}_{\mathrm{solid}}\right]=\left[{\mathrm{ZVS}}_{\mathrm{tot}}\right]–\left[{\mathrm{ZVS}}_{\mathrm{cyan}}\right]$$

The lowest value estimate of colloidal ZVS concentration [ZVS_colloidal,min_], is calculated under assumption that both dissolved cyclooctasulfur and polysulfides are in the equilibrium with rhombic sulfur.

$$\left[{\mathrm{ZVS}}_{\mathrm{colloidal},\min}\right]=\left[{\mathrm{ZVS}}_{\mathrm{cyan}}\right]-\left[{\mathrm{ZVS}}_{\mathrm{dissolved},\mathrm{total},\mathrm{calcd}}\right]$$

The highest estimate of colloidal ZVS concentration [ZVS_colloidal,min_] is calculated under assumption that concentrations of both dissolved cyclooctasulfur and polysulfides are equal to zero.

$$\left[{\mathrm{ZVS}}_{\mathrm{colloidal},\max}\right]=\left[{\mathrm{ZVS}}_{\mathrm{cyan}}\right]$$

Preparation of samples and analysis for quadruple sulfur isotope composition for sulfide, sulfate and zero-valent sulfur was performed according to [41].

### Conversion of sulfur species to silver sulfide for isotope analysis

Water samples for sulfide isotope composition analyses were filtered through a 0.4 μm Nuclepore Track-Etched Membrane filter within 6 hours of sampling with 20% zinc acetate preservation and frozen on dry ice. Filters with precipitate were boiled for 3 hours with 20 mL 5 N HCl in order to distill the acid-volatile sulfide (AVS).

BaCl_2_ solution was added to the filtrate in order to precipitate BaSO_4_. BaSO_4_ samples were reduced to H_2_S by boiling for 3 hours with 25 mL of a reagent prepared from 500 mL 36% HCl, 320 mL 57% HI, and 156 mL 85% H_3_PO_4_[80].

Zero-valent sulfur was extracted from 5–10 L aqueous samples with CHCl_3_ (3 × 1/50 v/v). Chloroform was evaporated on a rotor evaporator to 20–30 ml volume. Sample was transferred to the reduction reactor and remaining chloroform was evaporated under gentle flow of nitrogen. The zero-valent sulfur was converted to H_2_S by boiling for 2 h in a solution of 10 mL 12 mol L^-1^ HCl, 40 mL 99.98% ethanol, and 20 mL CrCl_2_ solution filtered through 0.20 μm filter [81]. CrCl_2_ solution was prepared by stirring of the mixture of 208 g CrCl_3_ · 6H_2_O, 120 g Zn and 400 mL 0.5 N HCl for two hours under a gentle flow of nitrogen.

Sediments and cinders were shaken overnight with methanol (c.a. 30 ml MeOH per 1 g of wet sediment) in order to extract zero-valent sulfur. Methanol was evaporated on a rotor evaporator; sulfur was extracted from methanol-water mixture with dichloromethane. Dichloromethane extract was dried with anhydrous calcium chloride and evaporated in the reduction reactor under gentle flow of nitrogen.

After extraction of zero-valent sulfur with methanol sediment was subjected to AVS distillation, followed by CrCl_2_ reduction [82]. This reaction was performed by boiling of sediment for 3 hours with 20 mL of CrCl_2_ reagent. This analysis accounts for non-zero-valent chromium-reducible sulfur, e.g. pyrite sulfur.

Hydrogen sulfide produced in all reactions was trapped in 5% zinc acetate solution. A portion of solution was used for measurement of concentrations, and to the rest of solution, silver sulfide solution was added in order to convert zinc sulfide to silver sulfide. Each Ag_2_S sample was aged for at least 1 week and cleaned with sequential washes of 200 mL Milli-Q water, rinsed in 50 mL of 1 M NH_4_OH overnight, then washed with 150 mL Milli-Q water and dried at 55°C.

### Quadruple sulfur isotope analysis

Conversion of silver sulfide to sulfur hexafluoride and analysis for quadruple sulfur isotope composition was performed according to [41]. Silver sulfide was quantitatively converted to SF_6_ by reaction with 10-fold excess of F_2_ gas at ~250°C for ~8 hours in Ni bombs. SF_6_ was purified cryogenically by distillation in an ethanol slurry at -115ºC, and by gas chromatography on a 12’ molecular sieve 5 Å/Hasep Q column with thermal conductivity detector. The isotopic abundance of the purified SF_6_ was analyzed on a Finnigan MAT 253 dual inlet mass spectrometer at m/e-values of 127, 128, 129, and 131 (^32^SF_5_^+^, ^33^SF_5_^+^, ^34^SF_5_^+^, and ^36^SF_5_^+^). Analytical uncertainties on sulfur isotope measurements, estimated from long-term reproducibility of Ag_2_S fluorinations, are 0.14, 0.008, and 0.20 (1σ) for *δ*^34^S, Δ^33^S, and Δ^36^S, respectively. Typical standard deviations between analyses of *δ*^34^S, Δ^33^S and Δ^36^S were 0.02–0.05‰, 0.01-0.02‰, and 0.1–0.2‰, respectively.

Isotopic measurements are reported on a VCDT scale, assuming the composition of IAEA S-1 is *δ*^34^S = -0.30‰, Δ^33^S = 0.094‰, and Δ^36^S = -0.70‰.

### Isotopic composition notations

We define ^3X^S isotopic composition of sulfur species in permil (‰) using standard delta (δ) notation:

(5) δ3XS=Rsample3X/RVCDT3X-1

where ^3X^R_sample_ is the isotopic ratio of a sample:

(6) $${}^{3X}R\;{=}^{3X}S{/}^{32}S\;\mathrm{for}\;3X=33,34,\mathrm{or}\;36)$$

where ^3X^R_VCDT_ is the isotopic ratio of the starting sulfide relative to Vienna-Cañon Diabolo Troilite (VCDT).

We define the fractionation between two sulfur species, A and B, as *δ*^34^S_A_-*δ*^34^S_B_.

The minor isotope compositions of sulfur species are presented using the Δ^3X^S notation, which describes the deviation of a sample datum in ^33^S or ^36^S (in ‰) from a reference fractionation line:

(7) Δ33S=δ33S-Rsample34/RVCDT340.515-1

and

(8) Δ36S=δ36S-Rsample34/RVCDT341.90-1

The exponents 0.515 and 1.90 are reference values assigned to approximate mass-dependent fractionations during thermodynamic equilibrium isotope exchange at low temperature [63,83].

## Competing interests

There are no competing interests.

## Authors’ contributions

The authors participated in the field work and writing of the paper. AK, JF and GD planned the research. AK, GD and ZFM performed samples analysis. All authors read and approved the final manuscript.
